# Hand constraint reduces brain activity and affects the speed of verbal responses on semantic tasks

**DOI:** 10.1038/s41598-022-17702-1

**Published:** 2022-08-08

**Authors:** Sae Onishi, Kunihito Tobita, Shogo Makioka

**Affiliations:** 1 Department of Sustainable System Sciences, Osaka Prefecture University, 1-1, Gakuen-cho, Naka-ku, Sakai, Osaka 599-8531 Japan; 2 Department of Psychology, Osaka Metropolitan University, 1-1, Gakuen-cho, Naka-ku, Sakai, Osaka 599-8531 Japan

**Keywords:** Human behaviour, Cognitive neuroscience

## Abstract

According to the theory of embodied cognition, semantic processing is closely coupled with body movements. For example, constraining hand movements inhibits memory for objects that can be manipulated with the hands. However, it has not been confirmed whether body constraint reduces brain activity related to semantics. We measured the effect of hand constraint on semantic processing in the parietal lobe using functional near-infrared spectroscopy. A pair of words representing the names of hand-manipulable (e.g., cup or pencil) or nonmanipulable (e.g., windmill or fountain) objects were presented, and participants were asked to identify which object was larger. The reaction time (RT) in the judgment task and the activation of the left intraparietal sulcus (LIPS) and left inferior parietal lobule (LIPL), including the supramarginal gyrus and angular gyrus, were analyzed. We found that constraint of hand movement suppressed brain activity in the LIPS toward hand-manipulable objects and affected RT in the size judgment task. These results indicate that body constraint reduces the activity of brain regions involved in semantics. Hand constraint might inhibit motor simulation, which, in turn, would inhibit body-related semantic processing.

## Introduction

Our knowledge of an object, such as a pencil, depends on what we implicitly know about body movements—in this case, how to grip the pencil and how to move our hands and fingers in a coordinated manner to write. At least part of our knowledge is embedded in the interactions between the body and objects and between the body and the environment. Based on this framework of embodied cognition, many studies have explored the relationship between word meanings and body motions^1–8^. It is becoming clear, through behavioral and neuroscientific studies, that word semantics are closely related to motor simulations. For example, several researchers measured reaction time (RT) after participants learned to map specific gestures to objects or words denoting the objects^9,10^. Faster responses were found when the mapped gestures were congruent with the gestures typically associated with the objects. This suggests that the actions compatible with the objects are automatically activated not only by the visual information of the objects but also by the semantic information of the words. Moreover, action verbs (e.g., “grasp”) followed by an object name elicit the formation of a “motor prototype” of the object^11,12^. These findings seem to imply that semantic processing related to object manipulation is embodied. However, others have poisted that these results can be explained by the view that conceptual and motor systems do not share processing resources^13^. Chatterjee^14^ pointed out that abstract semantic processing requires disembodiment. An alternative perspective is that these results are not due to embodiment but rather encoding of the object's size^15^.

To ascertain whether conceptual and motor systems share at least some processing resources, studies have employed physical constraints that prevent body movements. For example, hand immobilization was found to decrease the proactivity of gaze behavior when observing others grasping an object. Specifically, gaze shifts became slower when participants’ hands were tied behind their back^16^. Dutriaux and Gyselinck^17^ confirmed that conceptual knowledge of words is closely related to somatosensory and motor systems. The authors asked participants to learn lists of words for manipulable or nonmanipulable objects; then, the authors compared data from a condition in which participants had their hands behind their back while memorizing words with data from a condition in which their hands were unrestrained on the desk. In a later free recall task, performance on words denoting objects that can be manipulated with one’s hands was reduced by restraining the hands. They speculated that information about the impossibility of hand movement might have suppressed activity in brain areas involved in motor simulation, as a possible reason why hand restraint suppressed memory for objects that could be manipulated with one’s hand. However, under their experimental conditions, hand position and hand visibility were not controlled, so Onishi & Makioka^18^ examined the effect of posture and visibility in more detail. The authors found that only suppression of hand movements degraded the memory of words denoting manipulable objects, not the visibility or position of the hand (whether the hand was placed on a desk or folded behind the back).

It has been shown that the activity of corticospinal tracts while participants were imagining a specific movement with their hand was influenced by their hand posture^19^. Corticospinal excitability was enhanced when the actual hand posture was congruent with the imagined movement. The relationship between hand immobilization and brain activity has been investigated using a variety of methodologies: motor evoked potential^20–22^, resting motor threshold^23^, local blood flow and event-related desynchronization^24^. However, it remains unclear whether constraining the hands during semantic tasks affects the activity of brain regions involved in semantic processing. In addition, although it has been found that hand restraint reduces memory for words denoting hand-manipulable objects^17,18^ , it is unclear at which processing stage this effect occurs. By examining the effects of hand constraints on the speed of semantic processing and brain activity, we expect to obtain a clearer understanding of semantic processing for objects that can be manipulated by hand.

Previous neurological studies have repeatedly indicated that the premotor and parietal lobes involve object operations and object knowledge^25–27^^.^ Patients with damage to the parietal lobe or premotor cortex show impairment in their knowledge of manipulable objects and the movements needed to manipulate those objects^28^. It is known that damage to the left parietal lobe can result in ideomotor apraxia, which is the inability to perform an action according to verbal direction, and poor use of certain tools^29,30^. Chao and Martin^27^ found that the ventral area of the premotor cortex (BA 6) and left inferior parietal lobule (LIPL) are more strongly activated when individuals are observing or naming manipulable objects, such as pencils, erasers, and umbrellas, than when observing or naming nonmanipulable objects, such as animals, faces, and houses. The authors considered that the left parietal lobe processes information about visual features that characterize objects as tools, consistent with the finding that the anterior part of the intraparietal sulcus (AIP) of a monkey is activated when the monkey merely looks at graspable objects^26,31^.

It has been shown that the LIPL is related to the meanings of words. The LIPL, including the supramarginal gyrus (SMG, BA 40), is more strongly activated when word pairs presented in consecutive order are closely associated than when they are weakly associated^32,33^. Furthermore, it is known that patients with Alzheimer’s disease show reduced activation in the LIPL, including the angular gyrus (AG, BA 39), when judging categories of images and words compared with healthy seniors. The AG contributes to semantic processing, such as word reading, comprehension, and retrieval memory^34^. These findings support the view that the LIPL contributes to semantic processing^35^.

On the other hand, it has been revealed that the left intraparietal sulcus (LIPS), which is adjacent to the LIPL, is activated while observing an object-related action or while grasping or manipulating an object^36^; this activation is found to be related to motor intentions before executing a movement^37^. The LIPS is also known to be involved in spatial attention^38^ and spatial working memory^39^, suggesting that the area contributes to visual information processing required for object manipulation and spatial processing. At the same time, there is some evidence that the LIPS is activated even without visual information^40^: the area was activated while subjects classified words into categories. The findings from these studies suggest that the LIPS may also contribute to the comprehension of meaning.

Regarding the functional role of the LIPL on tool knowledge, researchers have proposed a framework where the SMG is involved in "action tool knowledge" and the AG is involved in "semantic tool knowledge"^41^. Action tool knowledge represents information about how to manipulate tools, and the SMG is involved in motor imagery^42^ through processing hand shapes and movements^43–45^. Semantic tool knowledge is information about the associative relationship of tools (what they are used with) and the functional relationship of tools (which category they are classified into functionally). It has been found that the AG, which contributes to semantic knowledge^35^, is also involved in semantic tool knowledge^46^. On the other hand, it has been known that there exists a multimodal object-related network in the occipitotemporal cortex that responds to both visual and haptic stimuli^47^.

Taken together, data from these studies suggest that the LIPS and LIPL are candidates for the areas responsible for semantic processing of hand-manipulable objects. The meaning of hand- manipulable objects might be represented in the LIPS and LIPL in association with the shape and movement of hands during manipulation. As noted above, hand constraint decreases the memory performance for hand-manipulable objects^17,18^. However, the inhibition of semantic processing itself and the change in brain activity caused by hand constraints have not yet been verified.

The aim of this study is to reveal whether hand constraint affects the activity of brain regions involved in semantic processing and whether it affects the speed of semantic processing. Specifically, we tested two hypotheses: (1) the activity of the LIPS and LIPL is reduced upon hand constraint while subjects execute semantic processing of manipulable objects, and (2) hand constraint delays semantic processing of manipulable objects. If these hypotheses are correct, they would suggest that the LIPS and LIPL conduct semantic processing on hand-manipulable objects and that such processing is inhibited by hand constraints; furthermore, they suggest that the semantic processing in the LIPS and LIPL is related to the motor simulation of hand movements. We therefore defined the regions of interest (ROIs) for this study as the anterior (AIP) and posterior (CIP) areas of the LIPS and the anterior (SMG) and posterior (AG) areas of the LIPL. Viewing hand-manipulable objects activates the precentral cortex^27,48,49^; such activity might be altered by hand constraint. Therefore, it would be beneficial to simultaneously record the LIPL and premotor cortex. However, in this study, the number of available channels were limited; thus, the measurement was limited to the LIPL.

**Figure 1:**
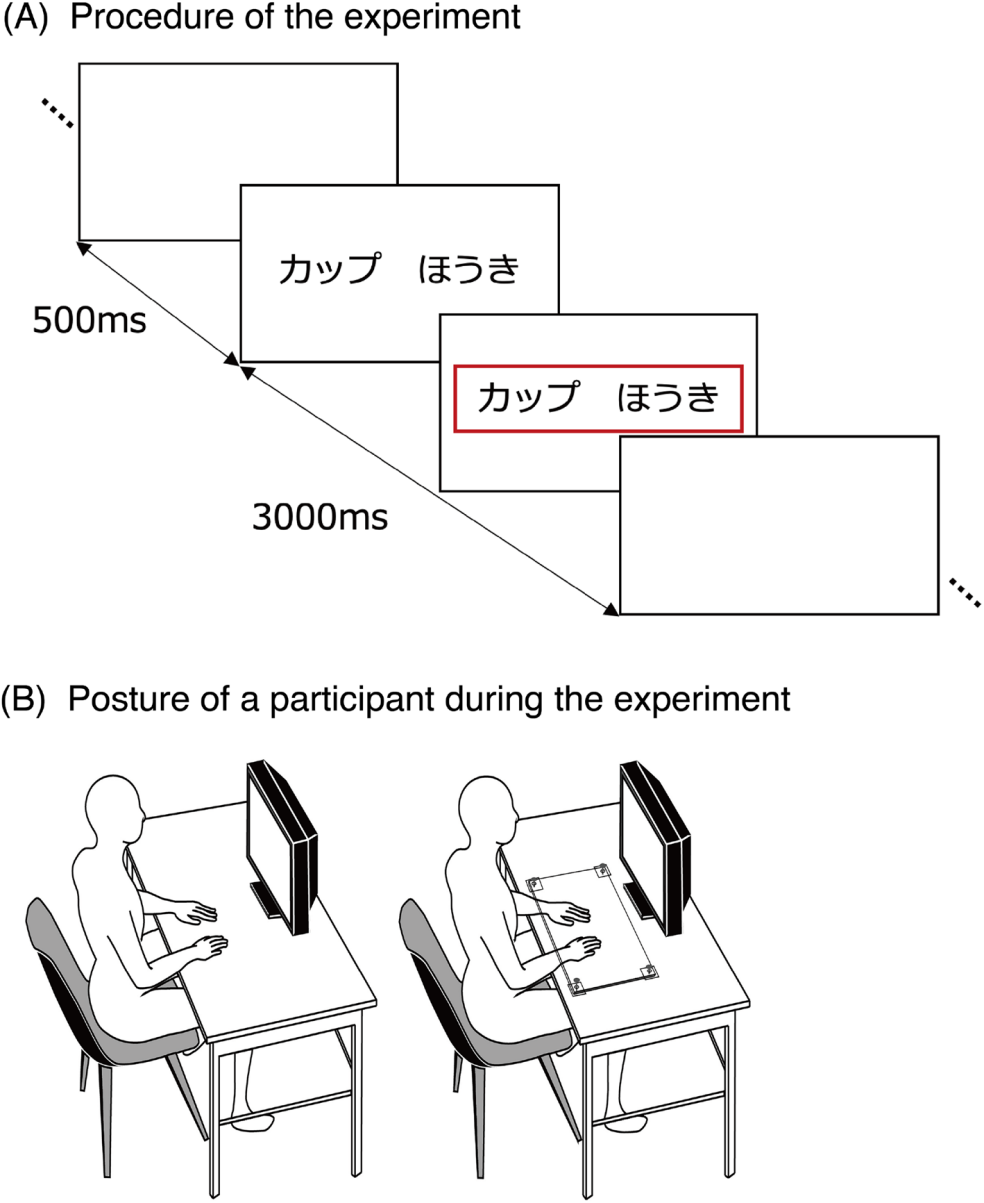
Experimental procedure. (**A**) Procedure of the experiment. Two words representing objects were presented on the screen, and the participants verbally indicated the larger of the two objects. In this example, the two words are cup and broom. A red frame appeared around the words after the voice response. After 3,000 ms elapsed from initial presentation of the words, a blank screen was presented, followed 500 ms later by the next pair of words. (**B**) Experimental conditions. Left: no constraint condition, right: hand-constraint condition. In the hand constraint condition, hand movements were restrained by a transparent acrylic board. To reduce participant fatigue, separate sessions for each condition were conducted on different days.

Because this research aimed to investigate the effect of hand restrictions, it is essential to allow the participants to engage in the task under very few physical constraints. Therefore, we measured brain activity by employing functional near-infrared spectroscopy (fNIRS) to ensure that participants could sit down and undergo the experiment with unconstrained physical movement. Participants were asked to judge the size of the object represented by the visually presented word. The stimulus words represented objects that could be manipulated by hand or objects that could not be manipulated by hand. We used a transparent acrylic board to restrain the hand movements of the participants so that they could place their hands on the desk and still see them, as was done in Onishi & Makioka^18^. This allows us to manipulate the constraint of hand movement independently of the position and visibility of the hand. As a behavioral measure, we also examined the effect of hand constraint on RT. The experimental procedure is shown in Fig. 1.

## Statistical analysis

### Naming latency

We conducted a repeated-measures analysis of variance (rmANOVA) with a 2 × 2 within-subjects factorial design; the mean RT on the size judgment task was the dependent variable and hand constraint (hand constraint or no constraint) and stimuli manipulability (manipulable or nonmanipulable) were independent variables. All independent variables were manipulated within participants. The significance level was set at 5% for all analyses. In the post hoc analysis, the false discovery rate (FDR)^50^ was calculated to control for the probability of type I errors given multiple comparisons; the FDR-corrected significance level was set at 5%.

### Hemodynamic activity of the ROIs

(a) The channel closest to the AIP (x = − 40, y = − 40, z = 40)^51^was selected among the designed probes (see supplementary information), and the z scores of the hemodynamic response of that channel were considered the dependent variable. (b) The channel closest to the CIP (x = − 15.7, y = − 67.8, z = 56.8)^51^ was selected among the designed probes, and the z scores of the hemodynamic response of that channel were considered the dependent variable. We conducted an rmANOVA with hand constraint and the manipulability of the stimuli as independent variables. (c) Two channels in the SMG (BA 40) and (d) two channels in the AG (BA 39) were selected among the designed probes, and the z scores of the hemodynamic responses of those channels were considered the dependent variables. We conducted an rmANOVA with channels, hand constraint, and manipulability as independent variables. The details of the fNIRS analysis are provided in the Supplementary Information.

## Result

### Naming latency

**Figure 2:**
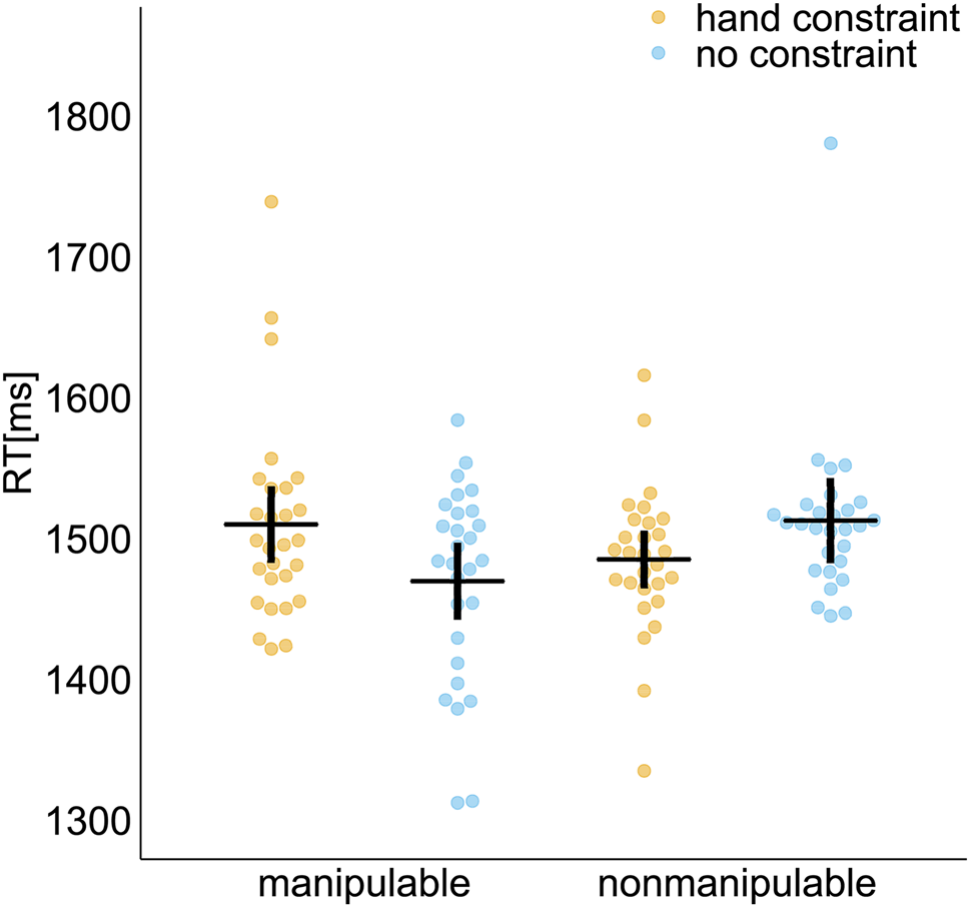
Mean RT (reaction time) in the size judgment task. The orange (hand constraint) and blue (no constraint) dots indicate the mean RT of each participant. Means are presented by horizontal lines. Vertical lines indicate the 95% confidence intervals calculated after subtracting the random effects of participants according to the Cousineau–Morey method^52,53^.

The mean RT for each oral response in the size judgment task is shown in Fig. 2. The number of trials in which participants failed to respond within the presentation time of the stimulus was 0.18% of all trials.

The results of rmANOVA and post hoc *t* test are shown in Table 1. The main effects of hand constraint and manipulability were not significant. The interaction between hand constraint and manipulability was significant. The post hoc analyses did not detect significant differences between conditions.

**Table 1:** The rmANOVA results of the mean RT (reaction time) and mean z score of each ROIs.

Mean RT (reaction time)	*F*(1,27)	*p*	*η*_G_^2^	
Hand constraint (H)	0.282	0.600	0.002	
Manipulability (M)	0.544	0.467	0.005	
H × M	5.630	**0.025**	0.067	*

Post hoc *t tests*	*t*(27)	Cohen’s *d*		
manip: hand const versus no const	1.724	0.572		
nonmanip: hand const versus no const	1.119	0.485		
Hand constraint: manip versus nonmanip	1.089	0.394		
No constraint: manip versus nonmanip	1.687	0.661		

(a) AIP	*F*(1,27)	*p*	*η*_G_^2^	
Hand constraint (H)	7.247	**0.012**	0.063	*
Manipulability (M)	0.077	0.783	0.001	
H × M	4.047	0.054	0.025	

Post hoc *t tests*	*t*(27)	Cohen’s *d*		
manip: hand const versus no const	**3.494**	0.992		**
nonmanip: hand const versus no const	0.007	0.168		
Hand constraint: manip versus nonmanip	1.032	0.343		
No constraint: manip versus nonmanip	0.886	0.310		

(b) CIP	*F*(1,27)	*p*	*η*_G_^2^	
Hand constraint (H)	4.675	**0.040**	0.039	*
Manipulability (M)	0.691	0.413	0.007	
H × M	5.911	**0.022**	0.015	*

Post hoc *t tests*	*t*(27)	Cohen’s *d*		
manip: hand const versus no const	**2.572**	0.691		*
nonmanip: hand const versus no const	0.131	0.142		
Hand constraint: manip versus nonmanip	0.496	0.081		
No constraint: manip versus nonmanip	1.375	0.433		

(c) SMG	*F*(1,27)	*p*	*η*_G_^2^	
Hand constraint (H)	2.956	0.097	0.017	
Manipulability (M)	1.917	0.178	0.008	
Channel (C)	0.671	0.420	0.004	
H × M	4.358	**0.046**	0.023	*
H × C	0.672	0.420	0.002	
M × C	0.085	0.772	0.000	
H × M × C	0.114	0.738	0.000	

Post hoc *t tests*	*t*(27)	Cohen’s *d*		
manip: hand const versus no const	2.081	0.682		
nonmanip: hand const versus no const	0.869	0.065		
Hand constraint: manip versus nonmanip	2.000	0.585		
No constraint: manip versus nonmanip	0.041	0.180		

(d) AG	*F*(1,27)	* p*	*η*_G_^2^	
Hand constraint (H)	2.722	0.111	0.022	
Manipulability (M)	0.048	0.828	0.000	
Channel (C)	1.353	0.255	0.004	
H × M	5.089	**0.032**	0.012	*
H × C	0.004	0.948	0.000	
M × C	0.567	0.458	0.001	
H × M × C	0.015	0.902	0.000	

Post hoc *t tests*	*t*(27)	Cohen’s *d*		
Manip: hand const versus no const	2.144	0.614		
Nonmanip: hand const versus no const	0.545	0.090		
Hand constraint: manip versus nonmanip	0.559	0.219		
No constraint: manip versus nonmanip	0.687	0.302		

This table presents the results of repeated measures analyses of variance on the mean z score of the hemodynamic response obtained over the duration of the target phase as well as the mean RT for each participant. FDR corrections were applied to the post hoc *t tests* to control for multiple comparisons.

Significant values are in bold.

*p < 0.05; **p < 0.01.

The significant interaction suggests that the interference effect of the hand constraint was stronger toward manipulable objects. Although the stimulus set used in the experiment was controlled for frequency and imaginability across conditions, the difficulty of the size judgments was not controlled. The mean RT was shorter in the manipulable condition without hand constraint, but this difference was not significant. However, significance of the interaction indicates that hand constraint had different effects on processing of manipulable and nonmanipulable objects, independent of the difficulty of judgments in each condition. These results are consistent with previous studies^17,18^, suggesting that hand constraint suppresses semantic processing of hand-manipulable objects.

### fNIRS results

We used oxygenated hemoglobin as the dependent variable because it is the most sensitive parameter of the hemodynamic response^54,55^. We calculated the z scores of the smoothed hemodynamic response in the target task by using the mean value and standard deviation during the last 5 s of baseline activity.

**Figure 3:**
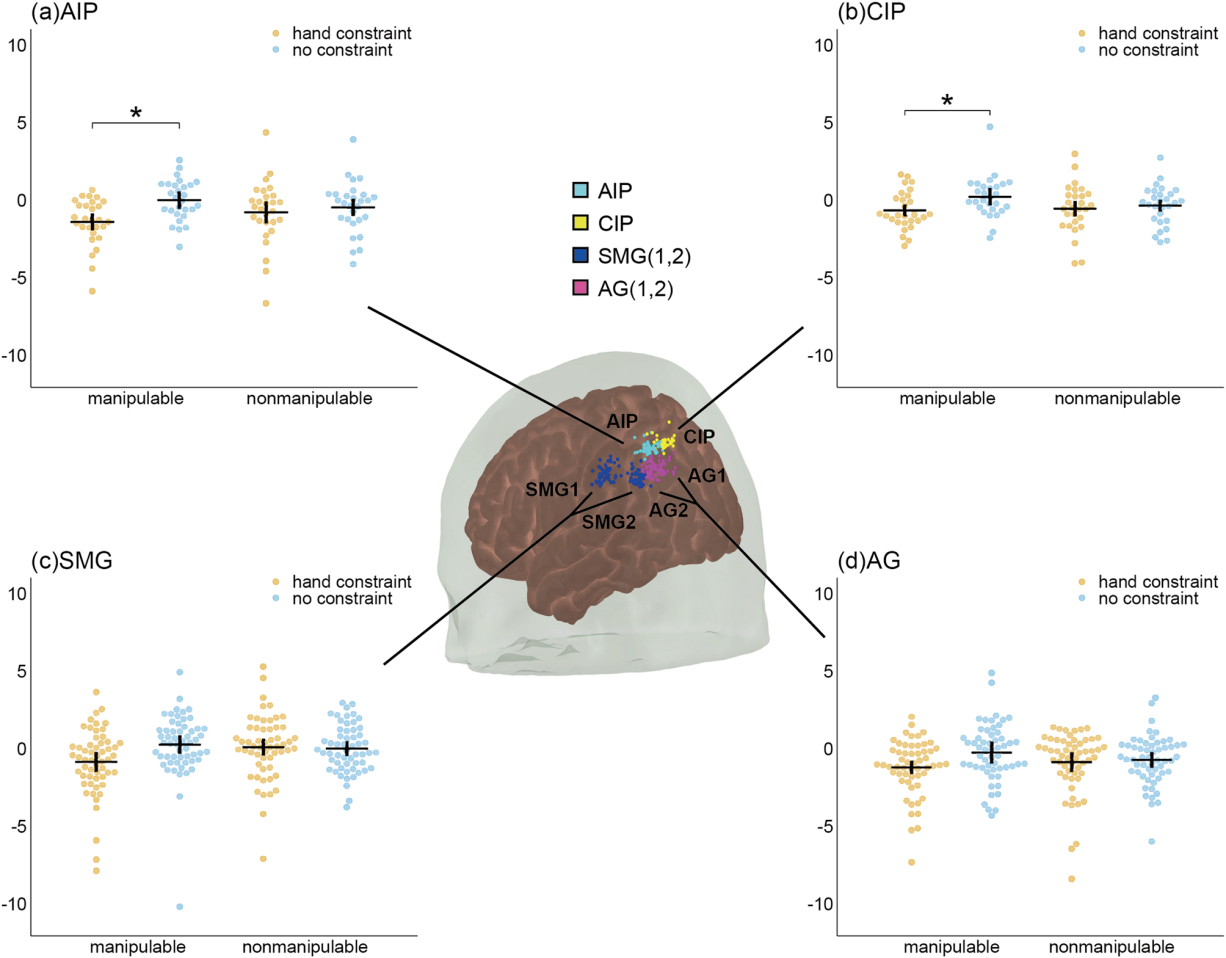
Hemodynamic activity while the participants were performing the size judgment task. Hemodynamic activity of the channels located in the (**a**) AIP (anterior part of IPS), (**b**) CIP (caudal part of the IPS), (**c**) SMG (supramarginal gyrus, BA 40) and (**d**) AG (angular gyrus, BA 39). Each participant’s mean z score of the hemodynamic responses in the size judgment task is shown. The orange (hand constraint) and blue (no constraint) dots indicate the mean z score of each participant's hemodynamic response during the task (n = 28). Means are presented by horizontal lines. Vertical lines indicate the 95% confidence intervals calculated after subtracting the random effects of participants according to the Cousineau–Morey method^52,53^. The color of each group of dots on the brain displays the positions of the channels of each participant in the Montreal Neurological Institute (MNI) coordinates. In SMG and AG, the data from two channels are displayed. FDR corrections were applied to the post hoc t tests to control for multiple comparisons. *p < 0.05.

**Figure 4:**
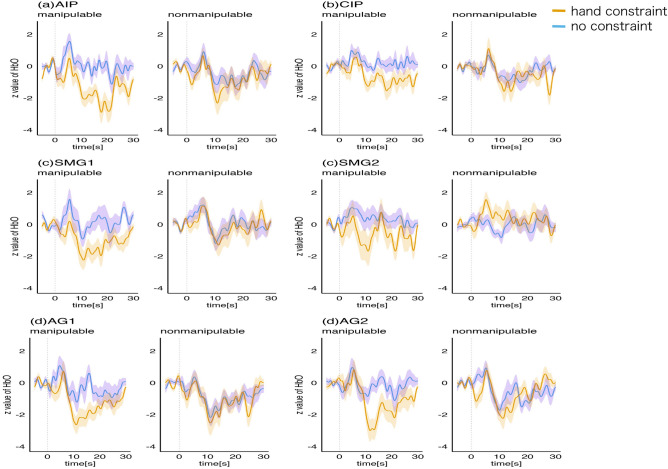
The averaged waveforms of hemodynamic responses in the LIPS and LIPL. The waveforms represent the mean z scores of the target task in each second. The standard error of the mean is illustrated as a shaded area around the waveform in corresponding colors. The task started at 0 s (vertical break line).

Figure 3 shows the mean z scores of hemodynamic responses, and Fig. 4 indicates the grand averaged waveforms of hemodynamic responses in the LIPS and LIPL. The dots on the brain displayed in Fig. 3 show the positions of channels that measure the activity of the AIP, CIP, SMG, and AG for each participant. The rmANOVA results showed a similar tendency for all regions (Table 1). The interaction between hand constraint and manipulability was significant in the CIP, SMG, and AG. In the post hoc analysis, the effect of hand constraint on manipulable objects was significant in AIP and CIP, while the main effect of hand constraint on nonmanipulable objects was not significant. The significant interaction in the CIP, SMG, and AG, together with the effect of hand constraint on manipulable objects in AIP and CIP, suggested that hand constraint suppressed the hemodynamic response to the manipulable objects in the LIPS and LIPL, which is consistent with the mean RT trend. This result is congruent with Chao & Martin's^27^ findings, showing that the AIP was activated by viewing and naming pictures of tools. It is also compatible with performance in memory tasks^17,18^.

## Discussion

The present study investigated how LIPS and LIPL activity during semantic processing of manipulable objects is affected by motor restrictions. The interactions between hand constraint and stimulus manipulability were significant in the hemodynamic responses of the CIP, SMG, and AG. In addition, the activity in response to manipulable objects was significantly decreased with hand constraint in AIP and CIP. Our first hypothesis that hand constraint reduces the activity of the LIPS and LIPL while executing semantic processing of manipulable objects was supported, at least for LIPS. As shown in Figs. 3 and 4, similar tendencies were observed between the AIP, CIP, SMG, and AG regions. The significant interaction in the analyses of the RT indicates that hand constraint had a more inhibitory effect on the manipulable object than on the nonmanipulable object. This result supported the second hypothesis that hand constraint delays semantic processing related to manipulable objects, and is congruent with the memory performance findings observed for manipulable objects when hand constraints were used^17,18^.

The current evidence that hand constraints suppressed semantic processing of manipulable objects in the LIPS and LIPL is consistent with previous studies suggesting that the LIPL provides essential motor information for recognizing manipulable objects^27^ and that the left parietal lobe has a key role in the use of tools and instruments^28,56,57^. This is also in line with several studies demonstrating that the LIPL is involved in word semantic processing^32,33,58^ and that the LIPS is activated in tasks that classify words into categories^40^. As mentioned in the introduction, the SMG involves processing motor imagery, such as hand shape and movement^41^.

Why did the hand restraints inhibit the activity of the LIPS and LIPL? As already mentioned, Dutriaux & Gyselinck^17^ speculated that information about the impossibility of hand movement might have suppressed activity in brain areas involved in motor simulation, resulting in decreased memory for manipulable objects. The authors assumed that the motor cortex is involved in motor simulation. On the other hand, we measured brain activity in the LIPS and LIPL and observed reduced activity due to hand constraints, suggesting that the LIPS and LIPL are engaged in motor simulation. It is known that sensory feedback that would occur during spontaneous action movement is predicted as the efference copy, and the predicted sensory consequences of the movement influence processing across the somatosensory cortex^59^. Several reports have suggested that the AIP compares the efference copy of a motor command with input sensory information about the action being performed^60,61^. Damage to the AG causes an inability to imitate gestures, which highlights the crucial role of the AG in encoding body parts^62^. The LIPL is further associated with the processing of somatosensory information. Specifically, the LIPL receives somatosensory information and processes spatial information, such as locations and distances obtained from tactile sensations, as well as structural information, such as body part location perceptions^63^. Since the somatosensory information that the hand is restrained is inconsistent with the results of motor simulations evoked by words denoting objects that can be manipulated with the hand, the activity of the LIPS and LIPL may be suppressed, and semantic processing based on the motor simulations may also be delayed.

The relationship between motor imagery and motor simulation has not been fully elucidated^64^. Understanding the meaning of words that denote hand-manipulable objects does not necessarily require the generation of motor imagery. However, in our experiment, participants were instructed to compare the sizes of two objects. This task may have evoked motor imagery of manipulating these objects by hand, possibly resulting in SMG activation.

In this study, the LIPS and LIPL, which are related to semantic processing, were defined as ROIs. Chao & Martin^27^ showed that the ventral premotor area (BA 6) is activated when a person is looking at manipulable objects, and it is quite possible that motor cortex activity is also decreased by hand constraint, as anticipated by Dutriaux and Gyselinck^17^. Previous studies have demonstrated that the premotor cortex is activated not only during actual movement but also when reading sentences containing motor actions^65–69^. Furthermore, some studies have suggested that the comparator system, which compares the efference copy with sensory feedback, is located in the premotor area rather than in the parietal lobe^70–73^. In future studies, the effect of hand restraint on motor cortex activity should also be examined.

We observed the effects of hand constraint on the speed of semantic processing and brain activity. Conversely, the influence of the stimuli’s semantic properties on the speed of hand movement responses has been investigated in previous studies. The motor-evoked potential in the hand observed when a transcranial magnetic stimulation (TMS) pulse was administered to the primary motor cortex was affected by the visual presentation of an adjective indicating that hand grasping is dangerous^74^, such as "sharp." When making judgments whether a visually presented noun referred to a natural or artificial object with either a precision or a power reach-to-grasp movement, adjectives that denoted decreased object graspability, such as "sharp," attached to the nouns, slowed RT^75^. In addition, RT to pictures of plants, animals, and artifacts, or words that represented them, were slower when the pictures or words implied that grasping them was dangerous^76–78^. These studies imply that hand movements are inhibited when a stimulus is meant to be dangerous to grasp with the hand. The effects of hand restraints on the speed of semantic processing and brain activity identified in this study are similar to these findings. Further insight into the relationship between semantic processing and motor simulation should be obtained by comparing the effects of hand constraint and stimulus danger on brain activity.

The present study shows, for the first time, that hand movement constraints suppress brain activity toward hand-manipulable objects and affected the RT of semantic processing. Because we adopted a design to analyze the activation of the whole block during the task, we could not analyze the time-related changes in the hemodynamic response in this study. Analysis of the temporal and causal relationships between the LIPS/LIPL and the ventral premotor cortex would reveal network structures of those areas. Future studies should examine the effects of body constraint on the flow of information across the semantic processing network in the brain.

### Method

#### Participants

We determined the sample size via G*Power^79^. Assuming a moderate effect size (f = 0.25), when "α = 0.05" and "1 − β = 0.8", the sample size required for a 2 × 2 within-subjects factorial design was 24, so we recruited approximately 30 participants. Thirty-six students and graduate students (24 females and 12 males, average age 19.94 years) at Osaka Prefecture University participated in the experiment. All participants agreed to wear the NIRS equipment, to maintain the designated posture during the test, and to have their oral responses recorded; they also provided written informed consent before the experiment. The experiment was approved by the Ethics Committee of the Graduate School of Humanities and Sustainable System Science, Osaka Prefecture University, and was performed in accordance with the latest version of the Declaration of Helsinki.

All participants were native Japanese speakers. With the exception of one non-respondent and two left-handed participant, all other participants were identified as right-handed. The data from the two participants who did not attend the second session and those from the six participants who displayed a hemodynamic response with excessive noise (see Supplementary Information) were excluded from the analysis, resulting in a total of 28 participants (mean age = 19.68 years, 18 females, 10 males, 25 right-handed).

### Materials

We used 64 names of hand-manipulable objects (e.g., cups, pencils) and 64 names of nonmanipulable objects (e.g., tires, stairs; see Supplementary Information Tables S1, S2). We defined manipulable objects as those that can be grasped and operated by hand. We selected the stimuli based on Amano and Kondo^80^ and Sakuma et al.^81^ to equalize the usage frequency, imaginability, and number of letters of each word between conditions. No significant differences were found between the conditions as a result of the *t test* (frequency of use: *t*_(126)_ = − 1.35, *p* = 0.18, number of letters: *t*_(126)_ = − 0.38, *p* = 0.70, mental imagery: *t*_(126)_ = 1.31, *p* = 0.19). Imaginability was scored as the difficulty of recalling the words as mental images^82^.

### Apparatus

All experiments were performed using MATLAB (version 2018a, The Mathworks Inc., Natick, MA, USA) and Psychtoolbox^83–85^ software version 3.0.17 running on an HP Pavilion Wave 600 with an Ubuntu 18.04.3 LTS operating system. Participants were presented with stimuli at a viewing distance of approximately 60 cm from the 24-in. monitor (ASUS VG248QE; resolution: 1920 × 1080). The stimulus words were an average of three characters in length, with each character approximately 1 cm × 1 cm in size.

### fNIRS data acquisition

We employed a 20-channel fNIRS system (LABNIRS, Shimadzu Corp., Kyoto, Japan), which is able to detect concentration changes in oxygenated hemoglobin (HbO), deoxygenated hemoglobin, and their sum by using three types of near-infrared light (wavelengths: 780, 805, and 830 nm). See Supplementary Information for details.

### Procedures

All participants wore the NIRS measurement holder on their heads. The holder was attached by the experimenter, who was careful not to make the participant feel any sense of compression. The participants were asked to keep their hands on the desk in the no-constraint condition and to hold their hands under a transparent board in the hand-constraint condition (Fig. 1). To reduce participant fatigue, separate sessions for each condition were conducted on different days. Each session took approximately one hour, including fitting of the holder and measurement of the channel position. In the hand-constrained condition, the height of the board was adjusted by the experimenter to the extent that the participants could pull their hands out if they pulled hard enough. The average interval between the 1st and 2nd sessions was 3.4 days. The order of the hand-constraint and no-constraint conditions was counterbalanced between participants.

After participants agreed to take part in the experiment, they engaged in eight practice trials. The experimental trials consisted of eight blocks. Each block consisted of a control phase in which participants performed morphological processing and a target phase in which participants performed semantic processing.

In the control phase, participants answered orally whether the nonword stimuli presented on a display were written in "hiragana" or "katakana". Japanese has two phonetic writing styles, "hiragana" and "katakana." All words in Japanese can be written in both hiragana and katakana; however, katakana is mainly used for foreign words. The task in the control phase can be regarded as comparable to the discrimination between uppercase and lowercase letters in English. Each stimulus was presented for 3000 ms at 500 ms intervals, regardless of whether the participant responded. A red rectangular frame was presented around the word only when the microphone obtained a verbal response. The control phase was conducted to obtain a baseline for the fNIRS analysis.

In the target phase, two words were shown on a display, and participants judged verbally which of objects denoted by the words was physically bigger than the other (Fig. 1). We instructed the participants that this task had no correct answer because object sizes are variable; then, we asked participants to judge the size based on their own images.

## Supplementary Information

Supplementary Information.

## Data Availability

The datasets collected in this study are available from the corresponding author on reasonable request.
