# Supplementary material for: Hand constraint reduces brain activity and affects the speed of verbal responses on semantic tasks
Source: Sci Rep. 2022 Aug 8;12:13545. doi: 10.1038/s41598-022-17702-1 (PMC9360433; doi:10.1038/s41598-022-17702-1)
Supplement: Supplementary file 1 — Supplementary Information. [file 41598_2022_17702_MOESM1_ESM.pdf]

Supplementary Information

Table S1. List of words denoting hand-manipulable objects

hand-manipulable objects				
word (Japanese)	word (parallel translation) (English)	frequency	number of characters	imaginability
鉛筆	pencil	1586	2	6.600
カップ	cup	13931	3	5.771
電池	battery	7111	2	6.057
ポット	pots	985	3	5.771
定規	ruler	156	2	5.286
ライター	lighter	3679	4	5.914
クリップ	clip	1953	4	5.567
封筒	envelope	1932	2	5.857
ビン	bottle	3086	2	5.514
鍵	key	6470	1	6.314
フライパン	frying pan	1088	5	6.514
うちわ	fan	704	3	6.314
フォーク	fork	4585	4	5.943
消しゴム	eraser	398	4	6.514
タオル	towel	3312	3	6.457
傘	umbrella	3532	1	6.143
つえ	cane	1422	2	5.343
マッチ	matches	3815	3	6.457
ボール	ball	27917	3	6.257
マイク	microphone	2785	3	5.886
電話	telephone	155535	2	6.314
バケツ	bucket	1132	3	6.429
ラケット	racket	662	4	6.029
歯ブラシ	toothbrush	642	4	6.571
筆	brush	3614	1	5.968
カメラ	camera	18134	3	6.629
付箋	sticky note	76	2	3.867
ドライバー	screwdriver	5039	5	5.613
ちりとり	dustpan	16	4	5.914
スコップ	shovel	564	4	5.629
辞書	dictionary	2858	2	5.743
ノート	notebook	8257	3	6.229
のこぎり	saw	192	4	6.429
絵の具	paints	1082	3	6.086
ハンドル	steering wheel	2252	4	6.086
ハンマー	hammer	2765	3	5.686
万年筆	fountain pen	624	3	5.943
ボールペン	pen	661	5	6.686
茶碗	bowl	507	2	5.714
キーボード	keyboard	976	5	5.886
チョーク	chalk	251	4	6.143
印鑑	stamp	1132	2	5.686
吊り革	strap	14	3	4.886
モップ	mop	147	3	5.943
電卓	calculator	342	2	5.686
コンパス	compass	254	4	6.200
輪ゴム	rubber band	171	3	5.913
本	books	908	1	6.200

折り紙	origami	668	3	6.229
せっけん	soap	1095	4	5.743
ピンセット	tweezers	89	5	5.971
洗剤	detergent	1849	2	5.657
じょうろ	watering can	52	4	5.714
トレー	tray	580	3	4.914
リモコン	remote	1016	4	5.914
箸	chopsticks	1451	1	5.686
綿棒	swab	119	2	5.857
ルーペ	loupe	216	3	4.714
ハンカチ	handkerchief	1452	4	6.543
カード	cards	22195	3	5.857
ほうき	broom	299	3	6.171
ハンガー	hanger	326	4	5.971
水筒	water bottle	304	2	5.200
缶詰	canned food	1256	2	5.857

Table S2. List of words denoting nonmanipulable objects

**non-manipulable objects**				
**word (Japanese)**	word (parallel translation) (English)	frequency	number of characters	imaginability
暖炉	fireplace	169	2	5.871
鳥居	torii	508	2	4.771
棚	shelf	3701	1	5.54
階段	stairs	5817	2	6.371
バス	bus	29370	2	6.543
テント	tent	4384	3	5.771
カーペット	carpet	595	5	6.057
ベッド	bed	5781	3	6.400
踏切	railway crossing	2353	2	5.543
電車	train	15566	2	6.286
噴水	fountain	531	2	5.543
水槽	fish tank	1239	2	5.171
ビル	building	29850	2	5.886
街灯	lamppost	365	2	4.829
飛行機	airplane	6172	3	6.171
風車	windmill	1049	2	5.686
黒板	blackboard	870	2	6.086
タイヤ	tire	4465	3	6.343
トラック	truck	14851	4	6.400
橋	bridge	10663	1	5.800
テレビ	TV	86686	3	6.686
ボート	boat	4003	3	5.857
水車	water wheel	302	2	5.257
扇風機	electric fan	815	3	5.714
池	pond	227	1	5.943
煙突	chimney	544	2	5.657
灯台	lighthouse	626	2	5.314
砂場	sandbox	225	2	5.600
プール	swimming pool	479	3	6.314
フェンス	fence	3076	4	5.314

浴槽	bathtub	1329	2	4.911
マンホール	manhole	368	5	6.029
エレベーター	elevator	3934	6	6.429
段ボール	cardboard	2685	4	5.714
机	desk	4290	1	6.229
ストーブ	stove	1209	4	6.086
鏡	mirror	4398	1	6.171
換気扇	ventilation fan	486	3	5.086
アンテナ	antenna	1716	4	5.771
ガソリンスタンド	gas station	1537	8	6.229
カウンター	counter	4539	5	5.257
スケートリンク	skating rink	196	7	5.686
絵画	painting	8309	2	5.086
線路	railroad tracks	4333	2	5.743
電柱	electric poles	1104	2	5.581
門	gate	3673	1	5.371
掲示板	bulletin	6137	3	5.171
コンビニ	convenience store	7099	4	6.257
ブランコ	swing	45	4	6.314
掛け時計	wall clock	40	4	5.143
エアコン	air conditioner	3567	4	6.235
ヘリコプター	helicopter	4132	6	6.429
バス停	bus stop	953	3	5.886
ガードレール	guardrail	562	6	5.600
障子	sliding door	578	2	5.257
畳	tatami mat	2076	1	6.000
ベンチ	bench	9086	3	6.029
メリーゴーランド	merry-go-round	28	8	6.200
トンネル	tunnel	7286	4	6.371
交番	police box	2189	2	5.371
プロペラ	propeller	689	4	5.600
百葉箱	instrument shelter	103	3	5.714
バルコニー	balcony	689	5	5.600
カカシ	scarecrow	164	3	5.857

fNIRS data acquisition

We employed a 20-channel fNIRS system (LABNIRS, Shimadzu Corp., Kyoto, Japan), which is able to detect concentration changes in oxygenated hemoglobin (HbO), deoxygenated hemoglobin, and their sum by using three types of near-infrared light (wavelengths: 780, 805, and 830 nm). The probes were arranged using the international ten-twenty electrode system (see Figure S1). Seven transmitter and receiver probes were used to cover the LIPS and LIPL.

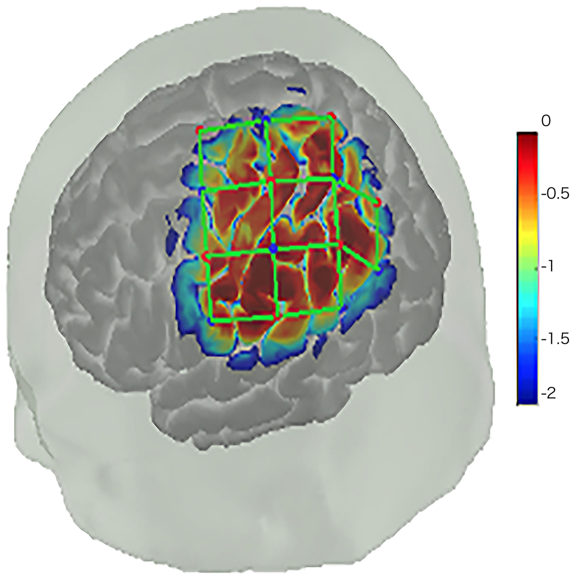

Figure S1. Probe placement**.**

Sources are displayed with red dots, detectors with blue dots and channels with green lines. The putative brain areas that are more sensitive to the array are visualized in hot colors on a standard brain template[A)].

Data preprocessing and analysis

Behavioral analysis

The oral responses of the participants were recorded. The RT for each trial was obtained based on the sound waveform[B)A)]. The response time was defined as the duration from the onset of stimulus presentation to the beginning of the sound waveform in each trial. The experimenter listened to the recorded audio, and sounds other than the participants' verbal responses were excluded from the analysis.

fNIRS analysis

Preprocessing was performed to compensate for artifacts and individual differences due to brain shape. We then analyzed the individual timeline data in each channel using a general linear model (GLM) that regressed the data into the hemodynamic response function (HRF), which models the temporal change in the blood flow signal reaching its peak following neural activity at five or six seconds after the stimulus presentation and converging to the baseline at approximately 20 s.

We preprocessed the data using MATLAB (2017b) and Homer3[C)]. AtlasViewer[C)] was used to identify the coordinates of each participant. Channels with low light intensity, i.e., a high noise level, were excluded from the analysis using the automatic light intensity setting of the source. None of those channels were used in this analysis.

The data acquired during this experiment contained a particular type of motion artifact, which was caused by the participants' jaw movements induced by the vocal response. The opening and closing of the mouth caused an abrupt displacement of the sources and detectors. We removed the artifact in the following method. Intensity data, which describe the amount of light emitted from a source position, were converted to optical density data, which represent the amount of

absorption through the brain tissue compared to the baseline[C)]. Wavelet filtering was performed to remove mainly spike noise using motion correction[C)]. This method decomposes the frequency components that consist of the temporally variable signal and removes anomalous wavelet coefficients considered motion artifacts. To achieve this, a probabilistic threshold is applied, setting to zero all wavelet detail coefficients that are assessed to be outliers of the distribution. After reducing the noise component, the wavelet coefficients are reconstructed into the original sequence by applying the inverse wavelet transformation.

To detect outliers, we used a threshold of 1.219 times (iqr = 1.219) the interquartile range, equivalent (assuming a Gaussian distribution of wavelet coefficients) to the $\alpha = 0.1$ threshold adopted in past studies[D)E)]. This preprocessing step is considered to be useful for removing motion artifacts existing in the raw data[D)F)].

Residual motion artifacts were identified as the portions of the signal exceeding a threshold in the change in amplitude (AMPthresh=5) and a threshold in the change in standard deviation (SDthresh=30) within a predefined time-window (tMotion=0.5 s). The points from the beginning of the window to a prescribed time (tMask=1) were regarded as motion artifacts. The data of six participants that were still contaminated by motion artifacts were discarded.

A bandpass filter (cutoff frequency: 0.00 and 0.05 Hz) was applied to the corrected optical density data mainly to remove the slow drift. Attenuation changes in all wavelengths (780 nm, 805 nm and 830 nm) were transformed to concentration changes in HbO using the modified Beer–Lambert approach (partial pathlength factor: 6.0[G)H)]) that partially takes into account the optical pathlength.

The mean hemodynamic response for each task block in each channel was recovered using a general linear model (GLM) approach. The time course of the HbO concentration change in the target phase was block-averaged using the hmrBlockAvg function of HOMER3 during the last 5 s of each control phase as the baseline. A set of Gaussian functions with a standard deviation of 0.5 s and with means separated by 0.5 s were used as temporal basis functions for the first 30 s after the start of the target task. The iterative weighted least square method[I)] was selected to solve the GLM matrix equation.

Coordinates for analyses

We used AtlasViewer, which is part of the Homer3 package. It performs probabilistic transformations from the positions in the electroencephalogram (EEG) 10-20 system to the standardized Montreal Neurological Institute (MNI) brain coordinates. Table S3 shows the channel positions of the designed probes in MNI coordinates and the average positions of all participants' probes in MNI coordinates. The positions of the channels of each participant, which were measured using a Polhemus FASTRAK Digitizer (Polhemus, Colchester, Vermont, USA) before the start of the session, are shown in Table S4.

Table S3. The estimated MNI channel positions of the designed probes at the ROIs and the mean positions of the channels for all participants.

	(1) **probe design**			(2) **average** (between subjects)			**Distance (mm)** (between (1) and (2))
	x	y	z	x	y	z	
**AIP**	-40	-55	45	-41.23	-53.98	47.66	3.10
**(sd)**				5.41	4.29	5.25	
**CIP**	-30	-59	49	-35.88	-61.13	50.09	6.34
**(sd)**				4.18	3.20	5.15	
**SMG1**	-43	-31	29	-52.32	-23.73	26.34	12.12
**(sd)**				6.64	3.33	4.59	
**SMG2**	-52	-52	31	-54.79	-49.73	24.36	7.55
**(sd)**				6.82	2.73	2.43	
**AG1**	-51	-62	40	-47.55	-54.77	30.80	13.21
**(sd)**				7.81	4.34	3.26	
**AG2**	-33	-62	40	-41.11	-58.55	30.16	12.20
**(sd)**				6.94	5.56	4.10	

Table S4. The estimated position of the channels in the MNI coordinates for each participant at the ROIs.

**Subject No.**	**SMG1**			**SMG2**			**AIP**			**CIP**			**AG1**			**AG2**		
	x	y	z	x	y	z	x	y	z	x	y	z	x	y	z	x	y	z
**1**	-47	-22	20	-61	-52	18	-38	-57	45	-34	-65	49	-45	-59	25	-44	-68	27
	-54	-23	30	-57	-52	25	-40	-56	50	-36	-64	53	-35	-50	26	-35	-60	28
**2**	-58	-27	28	-48	-51	24	-36	-60	50	-32	-63	47	-51	-68	32	-40	-67	31
	-51	-22	29	-61	-52	23	-44	-59	55	-34	-65	49	-55	-62	32	-35	-58	30
**3**	-65	-29	20	-45	-51	22	-45	-64	52	-34	-70	48	-53	-64	34	-36	-63	28
	-62	-19	32	-61	-52	23	-40	-56	49	-34	-61	49	-48	-58	29	-35	-56	28
**4**	-54	-27	31	-56	-52	26	-42	-58	52	-34	-61	49	-37	-52	30	-35	-57	29
	-54	-21	26	-50	-49	23	-44	-53	48	-39	-61	52	-44	-53	31	-53	-64	34
**5**	-44	-24	25	-54	-51	24	-28	-52	39	-33	-64	49	-45	-55	31	-47	-70	38
	-45	-24	28	-61	-52	23	-38	-52	43	-31	-60	44	-44	-52	25	-44	-63	25
**6**	-63	-32	23	-58	-56	23	-33	-57	42	-34	-66	47	-37	-52	30	-41	-68	27
	-45	-24	28	-47	-45	24	-40	-61	51	-32	-65	49	-49	-59	30	-35	-60	28
**7**	-54	-23	17	-63	-49	24	-44	-53	42	-30	-56	39	-38	-49	26	-35	-50	24
	-62	-21	26	-63	-50	26	-49	-56	52	-39	-61	52	-45	-49	29	-44	-57	29
**8**	-47	-24	20	-58	-50	20	-43	-56	44	-44	-68	49	-43	-52	26	-36	-53	28
	-52	-21	36	-63	-46	32	-29	-42	42	-42	-61	58	-56	-54	37	-46	-55	33
**9**	-63	-21	23	-58	-50	20	-43	-54	46	-32	-59	46	-39	-50	30	-36	-53	31
	-52	-29	22	-60	-53	22	-45	-62	50	-35	-66	49	-37	-52	30	-35	-58	30
**10**	-58	-27	19	-60	-52	23	-47	-59	47	-31	-60	44	-50	-57	31	-38	-59	25
	-48	-23	30	-62	-50	25	-43	-53	48	-39	-61	52	-57	-58	32	-37	-52	30
**11**	-50	-26	29	-48	-50	25	-28	-46	39	-36	-62	53	-42	-53	31	-35	-58	30

	-46	-29	24	-60	-52	23	-40	-57	46	-33	-60	46	-35	-50	26	-36	-60	27
**12**	-60	-27	22	-61	-52	22	-43	-52	49	-40	-61	54	-57	-58	32	-35	-50	26
	-47	-22	20	-61	-52	23	-39	-54	47	-34	-61	49	-39	-50	30	-35	-56	28
**13**	-45	-22	26	-54	-52	26	-28	-49	50	-24	-61	61	-37	-52	32	-38	-69	41
	-56	-20	20	-43	-46	26	-40	-50	46	-43	-59	54	-48	-52	33	-35	-50	26
**14**	-64	-17	32	-42	-42	23	-49	-45	59	-42	-54	63	-50	-51	32	-54	-57	38
	-53	-27	32	-48	-46	25	-37	-50	43	-40	-60	59	-55	-58	37	-51	-66	40
**15**	-57	-28	25	-61	-52	23	-41	-55	40	-30	-56	39	-44	-52	25	-38	-54	23
	-45	-24	28	-60	-52	23	-42	-57	51	-35	-62	51	-55	-62	32	-35	-56	28
**16**	-45	-23	30	-43	-46	26	-48	-53	57	-40	-60	59	-57	-56	36	-35	-54	32
	-54	-23	30	-57	-52	25	-40	-56	50	-36	-64	53	-35	-50	26	-35	-60	28
**17**	-63	-19	28	-63	-50	26	-38	-50	48	-40	-61	54	-57	-56	32	-43	-55	32
	-49	-22	24	-50	-49	23	-38	-50	38	-33	-57	42	-51	-56	31	-40	-56	25
**18**	-60	-21	32	-43	-46	26	-43	-52	49	-40	-61	54	-57	-56	36	-53	-60	36
	-47	-24	21	-43	-46	26	-40	-51	46	-39	-61	52	-54	-54	33	-53	-64	34
**19**	-49	-18	35	-61	-48	30	-49	-54	54	-41	-62	55	-48	-53	33	-39	-50	30
	-50	-24	29	-62	-50	25	-42	-51	43	-36	-59	45	-59	-56	30	-50	-58	31
**20**	-51	-21	27	-62	-50	25	-37	-51	39	-39	-61	47	-35	-50	26	-55	-64	32
	-43	-30	28	-49	-51	31	-38	-55	48	-37	-62	53	-54	-59	39	-43	-60	36
**21**	-48	-23	30	-49	-47	29	-43	-55	49	-40	-61	51	-46	-52	35	-35	-54	32
	-65	-22	24	-55	-51	23	-37	-51	41	-37	-61	48	-51	-56	31	-55	-64	32
**22**	-47	-24	24	-62	-51	25	-39	-55	48	-38	-64	54	-39	-50	30	-37	-52	30
	-55	-22	29	-43	-46	26	-43	-53	48	-36	-60	49	-51	-52	32	-35	-50	26
**23**	-48	-23	30	-55	-49	24	-49	-54	54	-36	-61	51	-48	-54	31	-45	-58	32
	-57	-21	21	-50	-49	23	-49	-54	54	-37	-61	52	-39	-50	30	-37	-52	30
**24**	-64	-17	32	-44	-41	25	-49	-43	60	-34	-50	54	-57	-51	37	-54	-56	38
	-47	-27	22	-47	-50	22	-45	-57	45	-37	-63	47	-55	-62	28	-37	-60	26
**25**	-39	-32	26	-51	-50	23	-34	-54	41	-27	-59	41	-57	-60	30	-37	-60	26
	-54	-25	23	-55	-49	24	-42	-53	45	-45	-64	52	-42	-55	29	-51	-68	32
**26**	-48	-23	30	-52	-49	23	-49	-54	54	-40	-61	58	-57	-57	32	-37	-52	30
	-52	-23	16	-61	-52	23	-43	-56	45	-33	-60	46	-38	-49	26	-40	-61	29
**27**	-50	-24	29	-60	-51	25	-46	-59	50	-33	-60	46	-55	-62	32	-51	-68	32
	-54	-25	24	-62	-50	25	-39	-51	40	-32	-57	41	-57	-58	32	-38	-54	23
**28**	-45	-24	32	-51	-50	27	-44	-59	56	-35	-61	51	-37	-52	30	-35	-57	32
	-45	-24	28	-54	-51	25	-47	-57	50	-32	-59	47	-57	-58	32	-53	-65	33
**AVERAGE**	-52.70	-22.73	26.71	-55.16	-48.18	24.46	-41.96	-51.98	47.04	-36.71	-58.86	49.27	-48.48	-53.04	30.77	-41.93	-56.88	29.80
**SD**	6.92	8.16	5.21	7.74	11.77	2.59	7.48	15.44	7.00	7.78	16.88	7.60	9.65	14.27	3.28	8.89	14.53	4.90

A) Collins, D. L., Zijdenbos, A. P., Kollokian, V., Sled, J. G., Kabani, N. J., Holmes, C. J., & Evans, A. C. (1998). Design and construction of a realistic digital brain phantom. *IEEE transactions on medical imaging*, **17**(3), 463-468.
B) Sakuma, N., Hoshimi,T., & Tatsumi, I.(1997). Measurement of naming latency of kana characters and words based on the speech wave analysis –Manner of articulation of a word－initial phoneme considerably affects naming latency-. *Neuropsychology*, **13**, 48-58.
C) Huppert, T. J., Diamond, S. G., Franceschini, M. A., & Boas, D. A. (2009). HomER: a review of time-series analysis methods for near-infrared spectroscopy of the brain. *Applied optics*, **48**(10), D280-D298.
D) Brigadoi, S., Ceccherini, L., Cutini, S., Scarpa, F., Scatturin, P., Selb, J., ... & Cooper, R. J. (2014). Motion artifacts in functional near-infrared spectroscopy: a comparison of motion correction techniques applied to real cognitive data. *NeuroImage*, **85**, 181-191.
E) Cooper, R., Selb, J., Gagnon, L., Phillip, D., Schytz, H. W., Iversen, H. K., ... & Boas, D. A. (2012). A systematic comparison of motion artifact correction techniques for functional near-infrared spectroscopy. *Frontiers in neuroscience*, **6**, 147
F) Molavi, B., & Dumont, G. A. (2012). Wavelet-based motion artifact removal for functional near-infrared spectroscopy. *Physiological measurement*, **33**(2), 259.
G) Boas, D. A., Dale, A. M., & Franceschini, M. A. (2004). Diffuse optical imaging of brain activation: approaches to optimizing image sensitivity, resolution, and accuracy. *NeuroImage*, **23**, S275-S288.
H) Delpy, D. T., Cope, M., van der Zee, P., Arridge, S., Wray, S., & Wyatt, J. S. (1988). Estimation of optical pathlength through tissue from direct time of flight measurement. *Physics in Medicine & Biology*, **33**(12), 1433.
I) Barker, J. W., Aarabi, A., & Huppert, T. J. (2013). Autoregressive model based algorithm for correcting motion and serially correlated errors in fNIRS. *Biomedical optics express*, **4**(8), 1366-1379
